# Crack Mitigation in Concrete: Superabsorbent Polymers as Key to Success?

**DOI:** 10.3390/ma10030237

**Published:** 2017-02-28

**Authors:** Arn Mignon, Didier Snoeck, Peter Dubruel, Sandra Van Vlierberghe, Nele De Belie

**Affiliations:** 1 Magnel Laboratory for Concrete Research, Department of Structural Engineering, Faculty of Engineering and Architecture, Ghent University, Technologiepark Zwijnaarde 904, B-9052 Ghent, Belgium; Arn.Mignon@ugent.be (A.M.); Didier.Snoeck@ugent.be (D.S.); 2 Polymer Chemistry and Biomaterials Group, Department of Macromolecular and Organic Chemistry, Faculty of Sciences, Ghent University, Krijgslaan 281, B-9000 Ghent, Belgium; Peter.Dubruel@ugent.be (P.D.); Sandra.VanVlierberghe@ugent.be (S.V.V.)

**Keywords:** hydrogel, autogenous shrinkage, freeze-thaw resistance, self-sealing, self-healing

## Abstract

Cracking is a major concern in building applications. Cracks may arise from shrinkage, freeze/thawing and/or structural stresses, amongst others. Several solutions can be found but superabsorbent polymers (SAPs) seem to be interesting to counteract these problems. At an early age, the absorbed water by the SAPs may be used to mitigate autogenous and plastic shrinkage. The formed macro pores may increase the freeze/thaw resistance. The swelling upon water ingress may seal a crack from intruding fluids and may regain the overall water-tightness. The latter water may promote autogenous healing. The use of superabsorbent polymers is thus very interesting. This review paper summarizes the current research and gives a critical note towards the use of superabsorbent polymers in cementitious materials.

## 1. Concrete and Its Problems

### 1.1. The Global Use of Concrete

Concrete is the most used man-made material with a world-wide production ranging from 35 up to 53 billion tons in 2014 (based on the cement production, equivalent to 8%–12% of the concrete production [1,2]). As such, it has become indispensable in our modern society. This extensive use can be explained by its high compressive strength, durability, relatively low cost and possibility to shape it into any form. As it is often combined with steel to create slim beams, columns or extremely long spans, concrete finds its application in floors and walls of buildings, in the infrastructure of bridges, roads, dams to even power plants but also in art applications in for example statues. It can be a material with a long service life, buildings from Roman times with “natural cements” can still be found standing today.

### 1.2. Ecological Disadvantages Accompanying Concrete

The popularity of concrete comes with a number of disadvantages. It brings an enormous ecological footprint. Approximately 5% to 7% of all CO_2_ emission can be traced back to the cement industry [3,4]. Indeed, for every ton of cement produced, approximately 1 ton CO_2_ is emitted due to the burning of limestone during which CaCO_3_ is converted into calcium oxide (CaO) by releasing CO_2_. On top of this, it is very water-consuming which poses a significant problem in for example countries with a hot-dry climate [5]. In the end, the disposal of concrete is also important as it contributes to a large part to the solid waste in industrialized nations. To address this issue, several solutions are possible including supplementary cementitious materials such as fly ash and silica fume to reduce the needed amount of Portland cement [1,5], recycled materials [1,5], reuse of wash water [5,6] and an improved durability and superior material properties of concrete [3].

### 1.3. Durability (and Sustainability) of Concrete

Durability has been a major issue in civil engineering, especially throughout the last 20 to 30 years. It may be defined as the ability of concrete to endure chemical attacks, weathering action and abrasion while maintaining the desired engineering properties [7]. Concrete deteriorates over time due to various time-dependent phenomena such as shrinkage, freeze/thawing, aggressive agents (such as sulfates and salts amongst others) and alkali-silica reactions. The criteria for safety and cost-effectiveness need to be fulfilled at all times. If not, maintenance, repair or even demolition will become necessary.

For these reasons, a life cycle cost analysis provides a superior indication of the actual cost compared to the initial cost associated with the construction. Furthermore, due to the long design working life (minimum of 50 years for conventional structures and 100 years for bridges and other civil engineering structures, according to NBN EN 1990), the production and disposal phase could be considered far less significant than the in-use phase. The major concern for concrete durability is rendering the cost minimal while still having a high performance [8,9]. This is not easy to assess, as the conditions where the concrete is exposed to and the concrete properties depend on the envisaged application. In structures such as tunnels or highways, concrete is in continuous service, meaning that regular inspection and maintenance of these structures is difficult and very expensive [8,10,11].

Concrete exhibits a low tensile strength despite its high compressive strength [12]. This needs to be compensated by an additional reinforcement to bear the tensile loads introduced by external loads, imposed deformations and expansive reactions [13,14]. Subsequently, cracking can occur and the crack width will need to be limited in view of the relevant exposure class and load combination. These crack width limitations can be found in Table 7.1N in the norm NBN EN 1992-1-1. The presence of cracks endangers the durability of concrete and can lead to corrosion of the reinforcement, since a pathway for harmful particles dissolved in fluids and gases is thereby generated [15].

Early-age cracking can be caused by drying and self-desiccation shrinkage. Fresh concrete may be subjected to cycles of shrinkage and expansion during the curing process which can lead to a differential stress [14]. This tendency results in potential crack formation. By curing the concrete, the evaporation of water can be prevented and the degree of cement hydration maximized [16]. High performance concrete has a low water-to-cement (W/C) ratio (<0.4) and will have an insufficient amount of mixing water for complete hydration. First, the capillary water is consumed, followed by a reaction of the cement with the more strongly bound gel water [17]. The microstructure densifies and this leads to reaction products which take up a smaller volume than the initial reactants and a lack of external water to fill up the voids, resulting in self-desiccation of the cementitious matrix and hence, shrinkage and cracks [16,18,19,20]. Plastic shrinkage occurs when the surface moisture can evaporate faster instead of being replaced by internal water, which causes the surface to shrink more compared to the bulk material. Autogenous shrinkage is the dimensional change of cement paste, mortar, or concrete caused by chemical shrinkage. At the time the internal relative humidity is below a certain given threshold (when additional water is no longer available), self-desiccation of the paste leads to a uniform volume reduction. The latter is not due to thermal causes, stress caused by external loads or restraints or due to loss of moisture to the environment [21]. Autogenous shrinkage can on the other hand also have a positive effect. It can rule out thermal expansion during the hardening and can ensure a favorable clamping pressure on fibers or aggregates embedded in the concrete. Nevertheless, cracking will still occur as early-age shrinkage develops when the cement paste has not reached sufficient strength. Mitigating the early-age cracking can be performed by reducing the overall cementitious content. By optimizing the aggregate gradation across the matrix, the cement paste required to surround the aggregates can be minimized. This is an autogenous strategy. Other examples include the use of saturated and fine lightweight aggregates, acting as internal water reservoirs for internal curing [16,18,22]. In this respect, difficulties can occur concerning the rheological consistency and especially with respect to a reduction of the concrete strength and elastic modulus. It is particularly helpful to mitigate autogenous shrinkage of concrete mixtures with a low W/C. Another example includes the use of a passive internal restraint system due to non-shrinking aggregates, resulting in a reduced shrinkage in the case of an increased aggregate volume [16]. However, for high performance concrete, this can lead to local stresses and thus still an occurrence of cracks as the stiffness of the paste comes close to that of the aggregates. A last autogenous strategy is the use of expansive cements which are either produced via the formation of ettringite or the hydration of free lime (CaO) or periclase (MgO). However, in this case, the expansion is very difficult to control as it depends on the distribution of the expansive components in the cement powder [23].

Cracking at later ages can be an effect of different causes. External restraints can play an important role in crack formation [24]. Other influences are sulfate attack, alkali-silica reaction, corrosion, differences in settlement, structural cracking due to corrosion of the reinforcements and/or overstressing the construction, tension cracking due to bending and thermally-induced cracking due to temperature changes, amongst others. One of the major cracking problems, i.e., pitting and spalling, is due to freeze-thawing. Due to the volume increase of water due to freezing, water inside the pore structure and cracks will start to expand, leading to stresses in the concrete material. One way of counteracting such volume increase is the use of an air entrainer. However, for transportation, this admixture is not stable. The trick is that a certain pore structure is created so that there is no scaling.

The formed cracks are aesthetically unappealing. In addition, the overall public perception when seeing cracks is that the structure has failed or is about to fail. However, when the crack is not structural, not too wide and is not leaking water, it can commonly be accepted. Therefore, one should engineer the material in such a way that is it acceptable for all parties.

In this review paper, superabsorbent polymers and the influence on the cementitious properties such as autogenous shrinkage, microstructure, mechanical strength, freeze/thaw resistance, water permeability and autogenous healing will be investigated.

## 2. Superabsorbent Polymers

**Figure 1:**
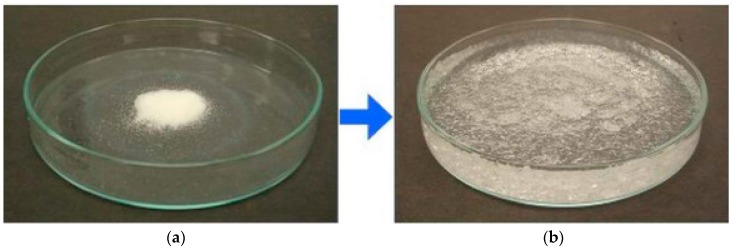
(**a**) Dry SAP powder and (**b**) swollen SAP.

Superabsorbent polymer materials (SAPs) are cross-linked hydrogel networks consisting of water-soluble polymers. SAPs generally compose of ionic monomers and need a low cross-linking density, to create a large fluid uptake capacity. SAPs can take up and hold aqueous solutions up to several hundred times its own weight [20,25,26,27,28,29,30,31,32,33] as can be seen in Figure 1, while retaining it even under pressure [25,28]. For the intended application in concrete, the swelling of the SAP is one of the most important characteristics. Stability of the SAP in the concrete matrix is also not insignificant, to the point that degradation needs to be minimized. Finally, the cost is on a practical level an important parameter. A minimization of the cost is necessary as large amounts (kg scale) will be needed.

**Figure 2:**
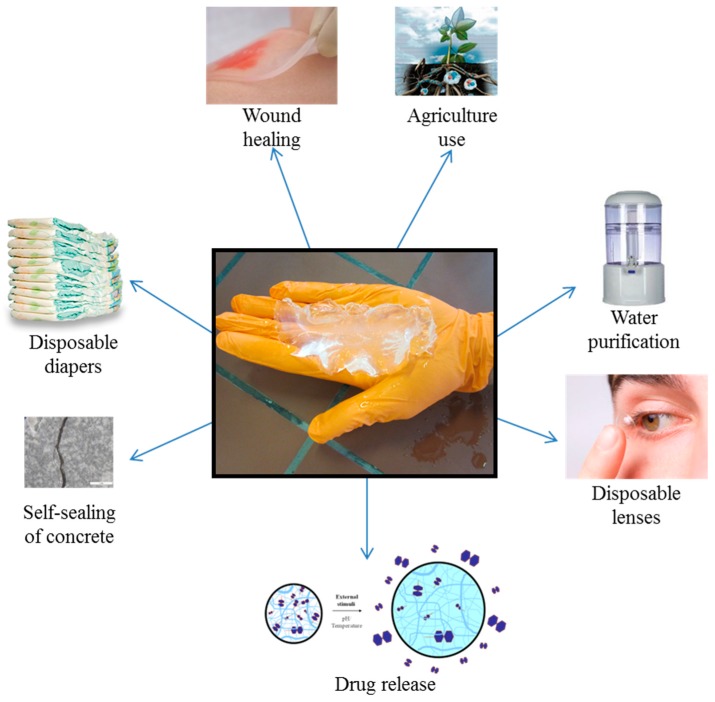
Overview of different applications of SAPs including diapers, biomedical (e.g., wound healing. Adapted from [57], with permission from © 2014 Kikgel), drug release [58] (World’s largest Science, Technology & Medicine Open Access) and disposable lenses, agriculture (Adapted from [59], with permission from © 2014 Terracottem), water purification and self-sealing and -healing of concrete.

SAPs are used nowadays in a plethora of applications, such as diapers [34], sanitary napkins [35,36] and biomedical purposes (e.g., disposable lenses, tissue engineering, drug release and wound healing) [36,37,38,39,40,41,42]. It can also have its purpose in the field of water purification [43] and as a water-blocking tape [35,44]. Another field of interest is the agricultural sector, for e.g., soil conditioners, nutrient carriers and water reservoirs applied to conserve water in dry areas [35,45,46,47,48,49]. A final application which gains more and more attention lately is the use of SAPs in mortar and concrete [50], and especially for self-sealing and self-healing of cracks [30,31,32,51,52,53,54,55,56]. An overview of the different biomedical and non-biomedical applications using SAPs is listed in Figure 2.

### 2.1. Natural versus Synthetic SAPs

SAPs can be classified in several manners: based on the absence or presence of charges (ionic, non-ionic, ampholytic or zwitter-ionic), the physical appearance, the presence of covalent or physical cross-linking bonds, but most importantly by the composition. The latter divides the SAPs between synthetic, semi-synthetic and natural SAPs [60]. Monomers used for synthetic SAPs are petrochemically-based and can include acrylic acid (AA), methacrylic acid (MAA), acrylamide (AM), dimethylaminoethyl methacrylate (DMAEMA), dimethylaminopropyl methacrylamide (DMAPMA), 2-acrylamido-2-methylpropane sulfonic acid (AMPS), etc. SAP networks can be created by using a synthetic cross-linker such as *N*,*N*′-methylenebisacrylamide (MBA). Additionally, by graft polymerization, synthetic monomers can be grafted on a natural backbone to create semi-synthetic SAPs [28,61,62,63]. Natural SAPs are based among other on polysaccharides or polypeptides. Polysaccharides can be obtained from biosynthesis occurring in plants and animals. Nowadays, polysaccharides produced by bacteria such as bacterial hyaluronan, gellan or xanthan are being investigated [64]. The currently used natural biopolymers for SAPs include alginate [64,65,66,67], chitosan [64,68], agar [69], carrageenan [70], dextrin [71], cellulose [47,72], starch [72], gellan gum [64,73] and proteins (from soybean, fish, and collagen-based) [60]. These materials are easily available, biocompatible, non-toxic and sustainable, making them a growing trend. As crude oil is becoming more expensive and finite [63,74], natural polymers become an cost-efficient and sustainable alternative. On top of this all, these polysaccharides possess functional groups such as amines, carboxylic acids and alcohols, which are easily adaptable for making derivatives useful for a variety of applications.

Mignon et al. [54,75] has developed modified alginate as a novel admixture for self-sealing and self-healing of mortar. For the same application, these alginates have also been tested with encapsulated carbonate producing bacteria [76]. To the best of our knowledge, the use of biopolymers for applications in mortar and concrete has only been reported scarcely. Nonetheless, these show great opportunities as they have proven to withstand high-pH cement filtrate solutions and are renewable [75]. Alginates can also be prepared at a reasonable cost. However, other polysaccharides such as agarose or chitosan will become too expensive to be used on a large scale.

Redox polymerization is the most used mechanism for the free radical synthesis. It proceeds at high rates at relatively low temperatures and are generally performed in aqueous solution, suspension or emulsion. Emulsion is the most appropriate technique to produce fine and regular particles sizes. However, the structure of polymer chains is less easy to control compared with bulk and solution polymerizations [77]. With bulk and solution polymerizations, an additional step of grinding the material is necessary, which will lead to more irregular particles compared to the emulsion polymerization. When the SAPs and thus macro pores in mortar or concrete will be irregular in shape, the compressive loads will not be transferred by dome action, which would be the case for spherical SAPs. This is one of the reasons why the compressive strength is lower for irregular shaped SAPs in systems with a high water-to-cement ratio [78].

An additional parameter of importance is the network density. Depending if an ionic or covalent type of cross-linking is used, a stronger gel will be formed in the latter case. A low cross-linking density will lead to a less brittle SAP, with a strong swelling capacity. A strong swelling capacity is needed for self-healing concrete [31]. On the other hand, strong swelling SAPs lead to larger macro pores which negatively influence the compressive strength [79]. It is often a combination of parameters which lead to the best results [80].

Acrylic acid (AA), partly neutralized by sodium or potassium salts, and acrylamide (AM) are the most used monomers in the industrial production of SAPs [29,81,82,83,84,85,86,87]. These monomers are considered to be non-renewable materials that are dependent on the petroleum industry. Acrylic acid is a colorless liquid. It has the ability to convert into its dimer. This spontaneous reaction is temperature dependent (an increase in temperature will lead to a stronger dimer formation). Water also accelerates this formation. The presence of this dimer must be minimized to limit yield reduction, loss of solubility, the occurrence of residual monomers, etc. To avoid this problem, manufacturers often perform moisture exclusion, last minute delivery and temperature-controlled storage [60,77]. The most often used technique to prepare acrylic-based synthetic SAPs is by a free-radical polymerization of the vinyl monomers in the presence of a multifunctional cross-linker [60,77]. Initiation is most often performed chemically by free-radical azo- or peroxide-based thermal dissociative species or through the use of a redox system [88]. A solution polymerization of AA in the presence or absence of its salts in an aqueous solution together with a water-soluble cross-linker such as *N*,*N*′-methylene bisacrylamide is a straightforward process which often leads to a gel-like flexible product [86]. Problems arising with this method include the lack of control over the reaction, the particle size distribution and the difficulty to handle a rubbery/solid reaction product. In an industrial setting, the inhibitor is usually not removed due to technical reasons [89]. Another polymerization technique to make these types of SAPs is the inverse-suspension polymerization. It is a highly flexible and versatile technique. A water-soluble initiator is used which typically provides superior efficiency over an oil-soluble type. When the initiator dissolves in the used dispersed aqueous phase, each particle contains the reactive species and behaves like a micro-batch polymerization reactor [83]. The inverse-suspension polymerization technique has been widely used for poly(acrylamide)-based SAPs due to its easy removal and management of the residual, hazardous acrylamide monomer from the polymer [90,91].

Some SAPs display reversible changes in swelling when exposed to physical, chemical or biochemical stimuli such as temperature, light or pH. These stimuli-based responses can be tailored by adjusting hydrogel composition, hydrophilicity of the network, shape, size and type/degree of cross-linking [88,92,93,94]. Therefore, there is a wide range of superabsorbent polymers, showing different behavior and thus different results in cementitious materials [30].

### 2.2. Stimuli-Responsive Superabsorbent Polymers

Some SAPs have the interesting ability to create large physical and chemical changes with only small environmental variations [95]. These ‘smart’ polymers [96] sense environmental stimuli [97] including changes in pH [93,97,98,99], temperature [97,100,101,102], light [103,104], pressure [105], etc. Drug release applications often use these pH-sensitive, ‘smart’ SAPs [97,106]. The goal is to release bioactive components at the correct time and/or appropriate site to precisely match physiological needs. The disease or injury causes a signal, which the SAP ‘senses’ and to which it responds accordingly [97]. Paul Flory [107] was one of the first who described these systems as Donnan membranes [108,109]. On the one hand, reactive groups in the polymer structure of pH-sensitive SAPs (e.g., carboxylic acid, sulfonic acid or amine groups) can repel or attract one another due to the formation of ions at specific pH-values. This is a parameter depending on the acidity or basicity of the environment. Identical charges repel one another and create more free volume where a higher amount of water can be absorbed and the swelling capacity thus increases. On the other hand, an equilibrium is formed of ion concentration gradients inside and outside the gel through osmotic pressure. Acid moieties will be negatively charged above the pK_a_ value, while this is the case for basic moieties below the pK_a_ value. Especially pH-responsive SAPs [30,31,97,106] could be extremely interesting for the envisaged application as admixture in mortar or concrete to mitigate autogenous and plastic shrinkage, to increase the freeze-thaw resistance and for the self-sealing and self-healing of cracks as will be explained in the upcoming sections.

## 3. Solving Crack Issues in Concrete

### 3.1. Tackling the Problem of Crack Formation: Autogenous Shrinkage

During the mixing process, these SAPs will take up a part of the initial mixing water, which at a later stage can be released inside the matrix during the hardening process thereby leading to internal curing and as such autogenous shrinkage reduction [16,50,51,110,111,112,113,114,115,116]. The SAPs can retain water and upon introducing them in mortar or concrete, result in water entrainment to reduce self-desiccation shrinkage during hardening [117]. The SAPs result in a similar effect as the LWA particles as they gradually discharge absorbed water and provide internal curing. However, it needs to be taken into account that only cracking due to restrained self-desiccation should be prevented to ascertain the cost and benefit of water entrainment. SAP percentages lower than 0.3 wt % compared to the added amount of cement are often used. A higher percentage of SAP could otherwise lead to changes in rheological properties and these effects cannot be neglected [114,118].

The effects SAP addition can have on the rheological properties of mortar depend on the W/C ratio, the presence and amount of superplasticizer and possible presence and amount of silica fume. This is an important parameter as it influences the compatibility, placement and ease of pumping [119] and the setting [120]. Adding SAPs equals removing available water from the matrix. If no additional water is added, the yield stress and plastic viscosity increases [121]. However, the effect SAPs have on concrete rheology has only sparely been described in literature [118,122,123,124]. A study made by Mechtcherine et al. [124] showed that the plastic viscosity depends on the initial uptake and release of the SAP. This is also related to the particle size, as a finer particle has more absorption surface leading to higher yield stress and plastic viscosity. An increase of the W/C ratio leads to higher absorption of the SAP and relatively larger rheological changes.

**Figure 3:**
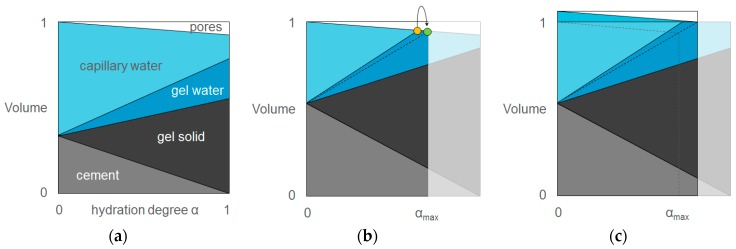
Principle of autogenous shrinkage in a system with a high water-to-cement ratio (**a**), and a low water-to-cement ratio without (**b**) and with internal curing (**c**).

The principle of autogenous shrinkage mitigation is shown in Figure 3. The figures follow the theory of Powers [125]. In Figure 3a, a system with a high water-to-cement ratio is shown. Here, a large amount of capillary water is available to receive complete hydration. In a system with a low water-to-cement ratio (lower than 0.42 [20,125]), the amount of capillary water is completely consumed (Figure 3b). The hydration continues with part of the bound gel water (curved arrow), but is limited. In case an additional source of water is provided (Figure 3c), no gel water is used and overall, the volume does not change and no autogenous shrinkage is found if the system adequately and ideally provides the water for internal curing.

**Figure 4:**
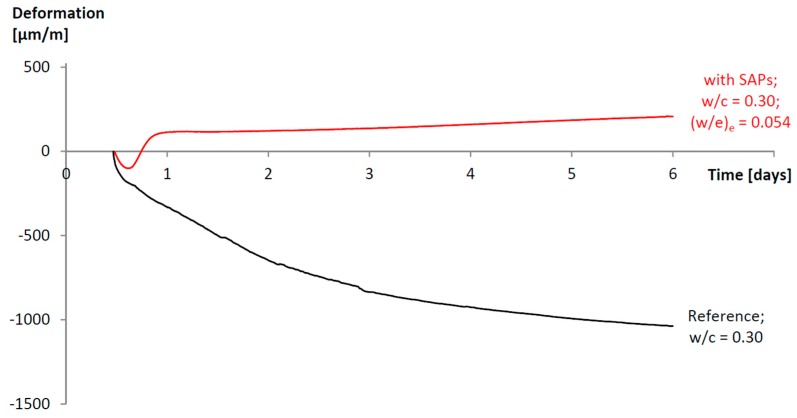
Mitigation of the autogenous shrinkage in a cementitious system with a water-to-cement ratio of 0.30 by means of superabsorbent polymer inclusion. Adapted from [17,78], with permission from © 2015 Elsevier.

An example of autogenous shrinkage mitigation is shown in Figure 4, redrafted after Snoeck et al. [17]. Here, a reference with a water-to-cement ratio of 0.30 is clearly showing shrinkage as the deformation is decreasing in time. By entraining an amount of water, equivalent to a W/C of 0.054 by the inclusion of superabsorbent polymers (bulk polymerized; approximately 100 μm in size), the shrinkage is completely mitigated in time.

There are several important factors to take into consideration to receive perfect mitigation of autogenous shrinkage. First, the cementitious mixture is an influencing factor. Autogenous shrinkage can already be mitigated in systems with Portland cement, silica fume [114] and in systems containing blast-furnace slag [17,126,127] or fly ash [17]. Second, the superabsorbent polymer is another factor of influence [78]. There are different shapes of SAPs, ranging from spherical and irregular to fibre types. The SAPs all show a different surface area available to give water towards the cementitious matrix for internal curing. This will give an influence. Furthermore, the polymeric properties of the SAPs are important. These influence the osmotic pressure responsible for swelling but also for desorption. If the SAP is releasing the water too fast (before final setting), then it would increase the total water-to-cement ratio. If it is too slow, it will keep its water and will not provide it to the cementitious matrix for internal curing. It is, therefore, of utmost importance to use specific types of superabsorbent polymers which can ideally give the water at the right times. If not, the autogenous shrinkage may not be reduced or counteracted and the overall properties at later ages may be completely different.

### 3.2. Influences on the Microstructural Properties and Moisture Transport Processes

As the available water (in mortar and concrete) influences the microstructural development and hardening, the water kinetics in samples with SAPs is a key parameter in the microstructural properties and moisture transport processes. Hydration of a mixture determines the microstructural development. Pastes with SAPs show less capillary porosity at later ages if additional water is mixed in to compensate for the decreasing workability by adding the SAPs (compared to if no additional water is used) [115]. The water released from the SAPs results in continued hydration, thus decreasing the micro-porosity at later ages [115], except from the macro pores created by the SAPs. An X-ray tomography study [128] showed a reduction of the amount of smaller capillary pores. This is due to two effects: (1) Filling of the existing pores with hydration products due to internal curing and (2) Reduction of the initial micro-cracks in the interior of a cementitious matrix, as autogenous shrinkage is partially reduced. Mercury Intrusion Porosimetry (MIP) [112,129] showed a higher total porosity due to macro pore formation in mortar or concrete specimens with SAPs and additional water. If no additional water was added, the total porosity was lower for mixtures with SAPs [129]. MIP does not directly measure the macro pores, since the range is narrow (working range of MIP; 0.1 nm < pore size < 100 μm), but macro pores do show up in the total porosity. The macro pores are hereby accessed only through smaller capillary pores, so that the volume is assigned to these narrower radii. Mixtures with the same effective water-to-cement ratio (ratio of the mixing water not held by the SAPs over the cement content), show the same capillary porosity [130]. The latter researchers also found a lower water permeability of cement pastes with SAPs and additional water (24 g water/g SAP based on the swelling capacity). The microstructure in between SAPs is denser due to internal curing and the macro pores do not interconnect. Therefore, the permeability is lower than of reference samples and this was also shown by using neutron radiography [56].

The effects in the micro (<2 nm) and mesopore (2–50 nm) range were studied by means of static and dynamic water vapour sorption tests [131,132]. The results show that cement pastes with SAPs and without additional water show a slight decrease in porosity in the micro- and mesopore range. Cement pastes with SAPs and with additional water show no significant change of porosity in the micropore range and a slight increase in the larger mesopore range.

### 3.3. Strength Decrease or Increase Due to Superabsorbent Polymers?

Microstructural properties directly affect the strength characteristics of the cementitious material. However, in the literature, the influences of SAPs are ambiguous. This influence can lead to an increase and/or a decrease in mechanical properties. It all depends on the used type of SAP, the addition of water to counteract the loss in workability and the mixing procedure, amongst others.

The flexural and compressive strength decrease when SAPs and additional water are added [30,112,113,114,115,133,134,135]. In these studies, additional water was added until the same flow/slump was reached as a compensation for the loss of workability due to the water uptake of SAPs compared to reference mixtures, unless stated differently. The lower the water-to-cement ratio, the more the strength of the composite is influenced by the addition of SAPs [115], and this effect is more pronounced at early ages due to the higher total porosity at early ages [112,136]. Internal curing leads to further hydration and the effect of SAPs on strength-loss is thus reduced at later ages. Theoretically, even a complete hydration due to internal curing is possible [125]. In Powers’ model, however, complete hydration in saturated systems is only possible in systems above a certain water-to-cement ratio (0.42 [20,125]) and this complete hydration is not possible for cementitious materials with a very low water-to-cement ratio. This is because one gram of cement chemically binds 0.23 g water and will bind 0.19 g of gel water [20]. The sum of both is 0.42 g. If this total amount of water is not available, total hydration is not possible unless water is provided from the surroundings during hardening.

Further hydration thus improves the mechanical properties but is mostly counteracted by the strength-loss caused by the SAPs [137]. SAPs thus have both a positive and a negative effect on the mechanical properties. A decrease in strength is observed at earlier testing ages (<7 days) while sometimes increases are obtained at later ages [110], especially in systems with supplementary cementitious materials where the internal curing reservoirs are available for the longer term pozzolanic reactions. In addition, the water-to-cement ratio has to be taken into account. At a value of 0.35 for example, the increased degree of hydration may counteract the strength loss due to macro pore formation [138]. At higher water-to-cement ratios, this is not the case.

The structure of a cementitious material is affected by the apparent water-to-cement ratio. As SAPs take up the mixing water, the apparent water-to-cement ratio appears lower, resulting in a closely-packed matrix and subsequent hydration due to the release of that water. Samples without SAPs do not have access to this free water. Therefore, water penetration in samples with and without SAPs is different. It is important to always take the effective water-to-cement ratio into account. One should make a verification when the matrix is hardened, i.e., by calculating the size of the actual macro pores and to back calculate the true absorption of mixing water [135,139].

### 3.4. A Way to Increase the Freeze/Thaw Resistance

According to different authors, there exists a link between freeze/thaw resistance and internal curing. As the SAPs will release their water during hardening, they will leave behind air-filled pores. During freeze/thaw cycles, these voids can protect concrete in a similar way as by using air entrainment [16,51,140]. In salt solutions, the SAPs swell less compared to demineralized water [30,78]. However, the swelling capacity is maintained during additional cycles of swelling [78]. With SAPs, the dimensions of the voids are fixed to the swollen state of the SAP during mixing, in contrast to regular air entrainment. Addition does not necessarily need to affect the compressive strength [141] and seem to be promising independent from local raw materials and production processes [142]. There was a considerable improvement in the material performance with the use of SAP in terms of decreasing mass loss due to scaling after the given number of freeze-thaw cycles [142]. The dynamic elastic Young’s modulus decreased less after tests without deicing salt. This demonstrates that increasing freeze-thaw resistance by use of SAP is a promising approach.

Another advantage is avoiding air void fusions during vibrations as these polymer particles are very stable in fresh concrete [143]. In a more practical point of view, concrete with air entrainment are less stable when transported. When using SAPs, the properties are fixed and the concrete can easily be transported. In addition, as the macro pore size is fixed, one can engineer the material in the ideal way, also taking the ideal spacing factor into account. The appropriate size-designed pore systems could improve the durability in terms of freeze/thaw resistance.

### 3.5. Sealing Cracks and Regaining the Water-Tightness of Structures

**Figure 5:**
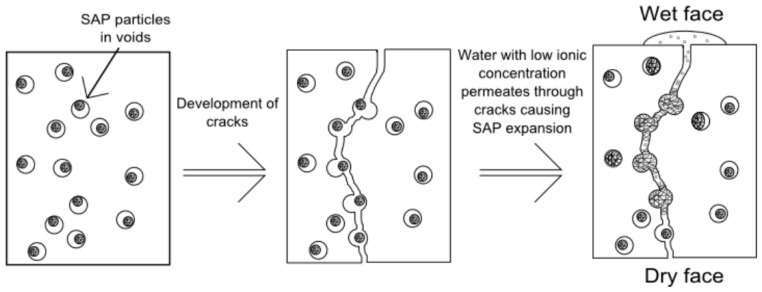
Schematic display of self-sealing of cracks in concrete by using SAPs. Adapted from [146], with permission from © 2010 Taylor & Francis.

As SAPs leave behind pores in the concrete matrix, these polymers can also be used for self-sealing and self-healing purposes [30,31,32,54,117,135,144,145]. Due to the presence of these voids, cracks will propagate through these weakened sections and ensure the SAP to be at the intended location. Water entering the cracks will fill the voids and result in SAP swelling, thereby resulting in expansion and preventing further ingress of any harmful particles as schematically described in Figure 5 [146].

**Figure 6:**
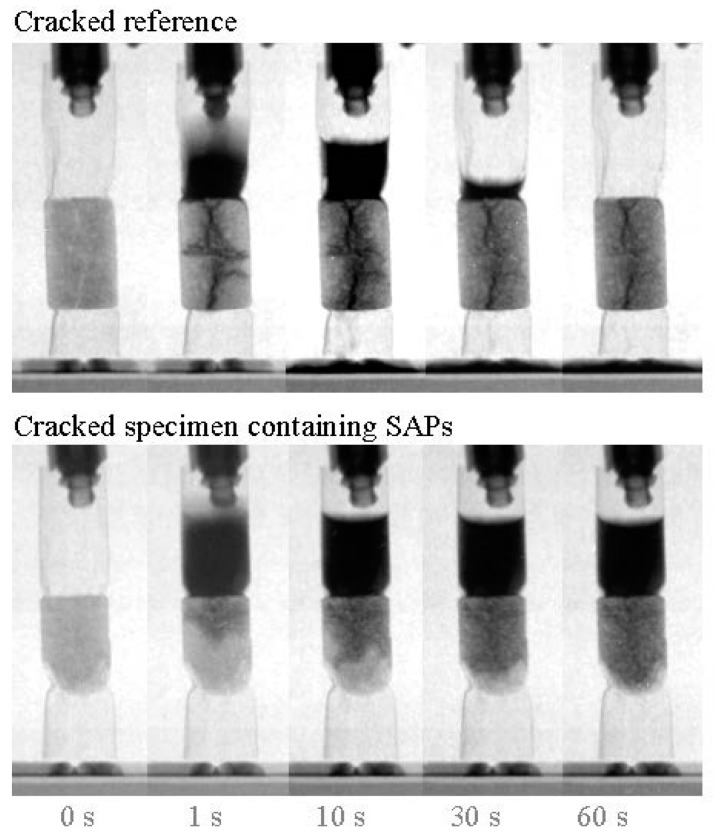
Decrease in water permeability in cracked mortar specimens due to the inclusion of superabsorbent polymers. Adapted from [56], with permission from © 2012 Elsevier.

When the crack is ideally sealed from further ingress, less deteriorating mechanisms may occur. The ingress of water for example could induce steel corrosion, frost attack, chemical attack and internal expansion, endangering the durability of a structure. Due to the (partial) prevention of this water movement, a more durable and sustainable material is designed. The sealing of a crack can be monitored by means of studying the (water) permeability. Due to their swelling capacity, upon contact with fluids, SAPs may cause a decrease in permeability of cracked cementitious materials as the swelling action is physically sealing the crack. Lee et al. [146,147,148] and Snoeck et al. [56,78,144] investigated the incorporation of SAP in concrete in order to obtain self-sealing properties. When liquids enter a crack, SAP particles along the crack faces will swell and block the crack. This is reflected in a decrease of water permeability through a crack. When a water head is imposed, the SAPs are able to withstand water movement, as studied and visualized by means of neutron radiography [56,144,149]. In Figure 6 the crack sealing capability of SAPs is shown. The top of the figure shows a cracked specimen and an imposed water head of 20 mm. It is clear that the water level decreases as a function of time. On the other hand, in the lower part of the figure, an analogous specimen but now containing SAPs is shown. As the SAPs swell, they seal the crack and the water head is maintained in time.

Song et al. [150] synthesized a superabsorbent resin in situ to repair concrete leakage, but this was rather a manual repair than an intrinsic sealing mechanism. The amounts of SAP sometimes are higher (up to 1 wt %, as is for promoted autogenous healing as well) compared to the amount needed for internal curing (typically 0.3–0.6 wt %).

### 3.6. Repairing Formed Cracks by Promoting Autogenous Healing

As a result of cracking, many concrete structures worldwide suffer from severe deterioration. Inspection, maintenance and repair will therefore become unavoidable and the restoration costs can increase up to approximately half the annual construction budget [151,152]. If cracks are formed in inaccessible places, manual repair even becomes impossible [30].

For crack repair, a variety of external techniques are available including manual repair using epoxy [153], polyurethane [13,154], coating the concrete surface by electro-deposition of chemical compounds [155], impregnation, replacement of contaminated concrete and steel bars as well as the application of coatings [156,157]. These solutions are expensive, time-consuming and in some cases, visually unattractive. Throughout the last decades, great advances have arisen in concrete repair technology. As such, instead of an external, passive and expensive treatment, an internal and active mitigation treatment can offer a superior solution. Self-healing materials have the ability to reverse the damage development once or multiple times and aid in expanding the lifetime and reliability of the concrete (i.e., the so-called ‘damage management concept’ introduced by Van der Zwaag) [158]. This is an enormous advantage for crack repair. Cracks would be able to seal and heal automatically, similar to broken bones and damaged skin or tissue that is able to regenerate.

**Figure 7:**
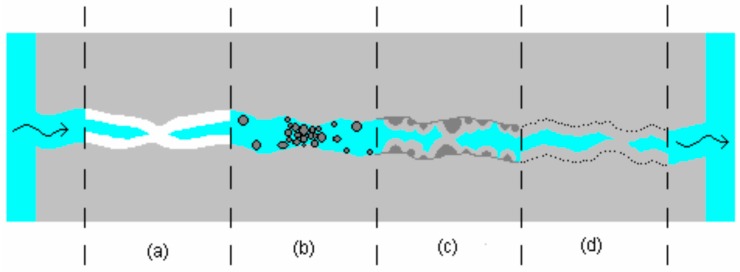
Different mechanisms of autogenous healing: (**a**) the deposition of CaCO_3_ or Ca(OH)_2_ crystals; (**b**) filling of the crack by pollutants in the water and loose concrete particles from spalling; (**c**) formation of new silicates by hydration of unreacted cement particles near the edge of the fracture and (**d**) expansive reaction of the hydrated cementitious matrix [163].

Concrete as such already shows some self-healing (i.e., autogenous healing) [159,160]. This effect has already been noticed in 1836 by the French Academy of Science [55,161]. The healing is believed to be the result of a combination of multiple mechanisms as indicated in Figure 7. Of these four causes, the primary mechanism with the highest self-healing capacity remains a matter of debate. In general, for very young concrete, self-healing can be attributed to further hydration due to the considerable amount of unhydrated cementitious materials. At a later age and in contact with water, Ca(OH)_2_ is formed and scattered along the crack edges. Subsequently, free calcium ions (Ca^2+^) react with bicarbonates (HCO_3_^−^) or carbonates (CO_3_^2−^) and the formation of CaCO_3_ becomes the dominant mechanism, whereby the crack is filled with a white crystallized substance [10,55,159,162].

Recent reviews on self-healing cementitious materials in general [117,164,165] and self-healing in strain-hardening cementitious materials [8,10] have been published. Further hydration and calcium carbonate crystallization will aid in healing small cracks completely up to 30–50 μm and partially up to 150 μm [144,162,166,167,168,169] in strain-hardening cementitious materials. The width of crack closure depends on the surrounding conditions and composition [170]. However, autogenous healing will not be sufficient for full crack repair in case of larger cracks and is very difficult to control [117]. As nowadays much finer cement particles are available, a reduction in the amount of unreacted particles can be noticed, which limits the potential of further ongoing hydration [171]. In the case of high strength concrete (50–100 MPa), due to a lower W/C ratio, there still is a high amount of unhydrated cement particles present. To obtain self-healing in these materials, further hydration of the grains is possible, in case external water is available. It is possible to increase the effect of autogenous healing. This is when three different measures including limitation of the crack width, the presence of moist environmental conditions and supply of certain chemical ions—the so-called building blocks—are performed.

As anticipated, a larger crack is more difficult to be sealed and self-healing will have an inferior effect [162]. The upper limit of crack width to enable crack healing depends on different test conditions used (e.g., mortar or concrete mixture, mix proportions, cement type, relative humidity, source of bulk water, etc.) [160]. The restriction in crack width may be 5–10 μm [172], 30 μm [32,78,169,173,174], 50 μm [175], 100 μm [176], 200 μm [159], 205 μm [177] or 300 μm [178] depending on the mixture composition and the test conditions. One way of limiting the crack widths, is by using (synthetic) (micro)fibers such as natural, glass, carbon, metal and synthetic fibers like poly(vinyl alcohol), polyethylene and polypropylene to receive strain-hardening cementitious materials [55,167]. These strain-hardening cementitious composites have also been developed to restrict crack widths to below 30–50 μm by mixing poly(vinyl alcohol) fibers due to multiple cracking [32,173,179]. The advantage over normal steel reinforcement bars is a uniform distribution throughout the matrix. Fibers can give more control over the crack propagation and width. Instead of a few large unhealable cracks, multiple smaller cracks will be introduced.

Self-healing of cracks is initiated by Ca^2+^, CO_2_ and unhydrated particles. Fly ash could be a way for autogenous healing. It is a pozzolan, which is a partly siliceous and partly aluminous material which has little cementitious value but which will react with Ca(OH)_2_ and water to form cementitious materials. A high percentage remains unhydrated even after 28 days. During crack formation, the water infiltrates and hydrates the fly ash in the presence of Ca(OH)_2_ from the pore solution to enable a CSH gel to deposit into the crack [180]. The healing products were mainly CaCO_3_, Ca(OH)_2_ [181,182] and calcium-silicate-hydrates (C–S–H) [183]. In addition, the latent-hydraulic blast-furnace slag can be useful [78]. All factors combined, i.e., unhydrated cement particles, formed Portlandite, free Ca^2+^ ions and pozzolans, will provide the necessary building blocks for healing when fluid water is available. However, the healing is limited (as water needs to be present) and needs to be stimulated. At 95% relative humidity (RH), there was no healing visible and it was concluded that the presence of water as curing medium was essential [184]. In a humid environment, so without the presence of liquid water, the material indeed does not show any form of healing [162]. To ensure a strong self-healing capacity, a humid environment is needed. Wet/dry cycles are appropriate. Another technique has been used by encapsulating water in paraffin. However, as the majority of the water leached out during the first days, the proposed methodology has not been viable so far [185]. To improve the contact with water, additional particles could also be incorporated such as lightweight aggregates (LWA) or superabsorbent polymers (SAPs).

LWA particles can reduce the autogenous shrinkage as these particles can act as internal water reservoirs and aid in providing internal curing [186,187]. A major disadvantage, especially associated with coarse LWA particles, is a decrease in the consistency and strength of the concrete matrix. Additionally, these aggregates replace normal aggregates which would have a higher strength [20,186].

SAPs thus would be very interesting to be used as a deliverer of fluids for promoted/stimulated autogenous healing. First of all, there is a distinction between self-sealing due to blockage and self-healing due to further hydration. This is important to know as a possible temporal self-sealing effect may not lead to a regain in mechanical properties. Due to the swelling of the SAPs, there is an immediate sealing, but not permanent. Together with the promoted autogenous healing, there will be a permanent sealing in time, when the healing products are formed.

**Figure 8:**
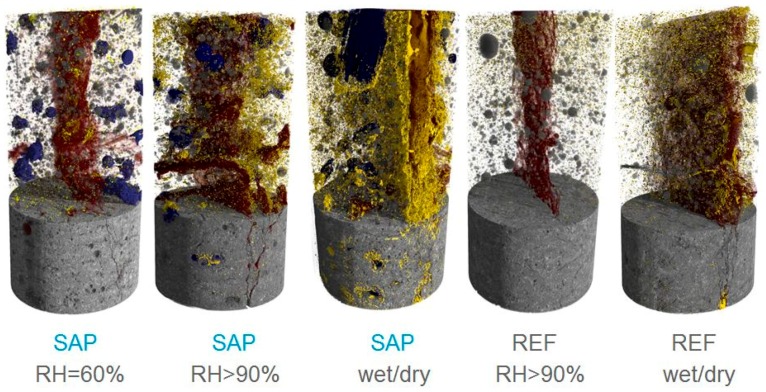
SAPs are able to stimulate and promote the precipitation of healing products in a crack, as was visualized by means of X-ray computed microtomography, redrafted after [190] (© 2016 Elsevier). Legend: grey concrete and pores, red crack, blue macro pores with superabsorbent polymers and yellow formed healing products after being stored for four weeks in a relative humidity of 60% (RH = 60%), more than 90% (RH > 90%) or in wet/dry cycles (1 h in water and 23 h in standard laboratory conditions) (wet/dry).

The use of SAPs to promote autogenous healing is dual. SAP particles swell due to mixing-water uptake during the mixing process and shrink during hardening of the cementitious material, leaving behind macro pores [188]. On the one hand, these macro pores act as initial flaws and (similar to the use of fibers) promote multiple cracking. On the other hand, the amount of SAP which can be added and size of the SAP is limited to minimize potential strength reduction. Secondly, SAP-particles are very useful for autogenous healing as these polymers absorb water during wet periods and slowly release it during dry periods. When liquids enter a crack, SAP particles along the crack faces will swell and block the crack. In addition, upon swelling, SAPs will initially seal a crack from intruding fluids, thus increasing the durability [146,147]. By absorbing fluids from the surroundings, water is available for healing [189]. This is beneficial as water will also be available during dry periods as well. This is preferential for CaCO_3_ crystallization after dissolution of CO_2_ from air in the water released from the SAPs. Formation of new cracks was noticed upon reloading the samples containing SAPs and regain of mechanical properties was shown. The regain was higher compared to reference specimens without SAPs. Even second reloading of healed samples leads to partial additional regain in mechanical properties [169]. Again, this regain is higher compared to the reference. The healing products were visualized by means of X-ray computed microtomography [190], as can be seen in Figure 8. In this figure, the concrete and some pores are shown in grey, the crack in red, the formed macro pores due to the SAPs in blue and the formed healing products in yellow. The extent of autogenous healing in a cementitious material depends on the crack depth. Only near the crack mouth (0 till 800–1000 μm) the crack is closed by calcium carbonate formation in case of wet/dry cycles [190,191]. In combination with superabsorbent polymers, the extent of healing was more substantial, as a high amount of precipitation—also in the interior—was found. For mixtures containing superabsorbent polymers there was even partial healing in the interior of the crack when stored at a relative humidity of 60% or more than 90%, especially in the vicinity of SAPs. As they are able to extract moisture from the environment [32,132], this small amount can be delivered to the cementitious matrix for promoted autogenous healing. Energy-dispersive spectroscopy combined with microscopic analysis showed that the healing products were mainly calcium carbonate.

The SAPs, as one of the promising paths for self-healing, were also applied in a real-scale concrete element (150 mm × 250 mm × 3000 mm) [145]. Several beams were made, with and without self-healing properties. Based on the measured crack width reduction over time, it was shown that best crack healing was obtained when superabsorbent polymers were added to the mixture.

The cementitious material with superabsorbent polymers is thus an excellent material to use in future building applications as the healing capacity is improved.

## 4. Challenges to Overcome When Using SAPs in Cementitious Materials

SAPs take up mixing water when added to mortar or concrete. If no additional water is provided, the workability and the effective W/C factor of the mixture will be reduced [143]. For example, 0.4 wt % SAP relative to the cement weight with an absorption capacity of 15 g/g can lower the effective W/C by 0.06 [114]. Both the swelling capacity of the SAP in a cement pore solution and flow measurement can provide an idea on the precisely required amount of supplementary water to eliminate the influence on the workability and obtain a similar W/C factor as the reference [135]. This needed amount depends on the polymer elasticity, the osmotic pressure in the mix and the polymer affinity for the mixing water which is more restricted in a concrete mixture than in cement filtrate solution. The solid particles limit the expansion of the SAPs [146].

SAPs can be mixed in in different ways. Most researchers dry mix the polymers prior to water addition. This ensures a homogenous distribution of the SAPs. Other researchers pre-saturate or pre-soak the SAPs prior to mixing [192,193]. Depending on the wanted application, this can be useful. However, in the experience of the authors, such pre-saturation will lead to clusters of SAPs, and a non-homogenous mixture. This is unwanted as the overall properties may differ and less control is achieved.

Different type of SAPs may influence the cementitious properties in an own intrinsic way. This can have wanted and unwanted influences. One needs to be very careful when selecting a specific type of SAPs as the wanted effect may not be attained. Additional parameters to take into account are also the cement alkalinity, aggregate chemistry and size, quality of the water used, batching procedures. The effect of the SAPs can serve one purpose but not the other. There thus are different applications where a specific SAP may be more useful compared to another. The SAPs thus take up additional water when mixed in and release it again due to a lower relative humidity during the cement hydration process. This hereby promotes internal curing and helps to maintain the internal relative humidity of the matrix [17]. By releasing this water, SAPs leave behind macro pores which negatively influence the strength. Despite this decrease in strength, a decrease in the micro-porosity can be noticed due to further hydration. The latter also causes a reduction in autogenous shrinkage and much less micro-cracks [135]. The important parameter to indicate whether the effect of further hydration and internal curing, leading to a strength increase, is more profound than the pore formation and strength loss on the other hand [138,194] depends on the added amount and size of SAPs. It is thus of the utmost importance to limit SAP swelling in fresh concrete, but not at the expense of the further hydration. As a solution, the use of pH-responsive SAPs, which will swell when needed, can become extremely useful [30,31]. Indeed, the high presence of cations in these cement filtrate solutions are also a point of attention, as they will cause a strong decrease in swelling, especially with the presence of the carboxylate moieties in the pH-responsive SAPs. However, this could also be turned to an advantage. Systems with acrylate moieties will absorb less water during the mixing in mortar and lead to smaller macro pores. On the other hand when cracks occur and water infiltrates the crevices, due to osmosis, the concentration of concentration of cations inside and outside the SAP will go to an equilibrium, as such increasing the swelling of the pH-responsive systems and making them more useful for the intended self-healing applications. This has already been tested for acrylate-based semi-synthetic SAPs based on methacrylated alginate combined with acrylic acid [75] but should definitely be a subject of further investigation.

## 5. Conclusions

Concrete is used on a world-wide scale. However, there are issues regarding its possible cracking behavior due to shrinkage, freeze/thawing and/or structural stresses. This crack formation can be counteracted by using superabsorbent polymers (SAPs) for different applications in concrete. SAPs already have shown being useful in diapers, biomedical applications, agricultural use, etc. In this review, the interesting properties of synthetic SAPs have been discussed, as well as the usefulness of natural SAPs. On top, the value of ‘smart’ pH-responsive SAPs is deepened. SAPs can be deployed for different applications in concrete such as reduction of autogenous shrinkage, increasing freeze/thaw resistance and inducing self-sealing and -healing of cracks.

When incorporating SAPs, the autogenous shrinkage is reduced and even completely mitigated. This is due to internal curing. The latter is beneficial in terms of strength properties, but on the other hand the overall formation of macro pores by the SAPs implies a negative influence on the strength, especially when using high water-to-cement ratios.

The freeze/thaw resistance is increased as the formed microstructural system of macro pores resembles the one of an air-entrained concrete. As the SAPs swell in cracks within hardened concrete, the SAPs decrease the water permeability and cause the so-called self-sealing effect. This is very interesting for constructions which need to be watertight. Another positive effect of SAPs is the promotion of autogenous healing. Even in standard laboratory conditions with a certain relative humidity, the specimens with SAPs show a regain in mechanical properties. In wet/dry cycles, the healing reaches values of 100% of the initial strength. Cracks are visually closed and this approach seems very promising to reduce the overall repair costs needed when concrete is cracking.

The typical amount of SAPs for mitigating autogenous shrinkage or to increase the freeze-thaw resistance is in the order of magnitude of 0.3–0.6 wt % (mass percent by cement weight). For self-sealing and self-healing, this amount is approximately 1 wt %.

However, certain challenges with SAPs in concrete still need to be overcome such as the need of additional water to compensate for the swelling of SAPs, the formation of SAP clusters and the negative effect due to the formation of macro pores. ‘Smart’ SAPs can offer a possible solution for a few of these problems. If one aims for self-sealing and self-healing, and not internal curing, one may use the pH-responsive SAPs. These SAPs will not swell upon mixing but will upon water ingress through a crack. Finally, it can without doubt be concluded that SAPs show great advantage to be used in concrete applications if applied in the correct way.

## Abbreviations

AA	Acrylic acid
AM	Acrylamide
AMPS	2-acrylamido-2-methylpropane sulfonic acid
CaCO_3_	Calcium carbonate
CaO	Calcium oxide—Free lime
Ca(OH)_2_	Calcium hydroxide—Portlandite
CO_2_	Carbon dioxide
C-S-H	Calcium-Silicate-Hydrate(s)
DMAEMA	Dimethylaminoethyl methacrylate
DMAPMA	Dimethylaminopropyl methacrylamide
LWA	Light-weight aggregate
SAP(s)	Superabsorbent polymer(s)
m%	Mass percent of cement weight
MAA	Methacrylic acid
MBA	*N*,*N*′-methylenebisacrylamide
MgO	Magnesium oxide—Periclase
W/C	Water-to-Cement ratio
